# Sequential combined test, second trimester maternal serum markers, and circulating fetal cells to select women for invasive prenatal diagnosis

**DOI:** 10.1371/journal.pone.0189235

**Published:** 2017-12-07

**Authors:** Paolo Guanciali Franchi, Chiara Palka, Elisena Morizio, Giulia Sabbatinelli, Melissa Alfonsi, Donatella Fantasia, Giammaria Sitar, Peter Benn, Giuseppe Calabrese

**Affiliations:** 1 Department of Medical, Oral and Biotechnological Science, Chieti-Pescara University, Chieti, Italy; 2 Department of Hematology, Pescara Hospital, Pescara, Italy; 3 Department of Genetics and Genome Sciences, University of Connecticut Health Center, Farmington, CT, United States of America; Montana State University Bozeman, UNITED STATES

## Abstract

From January 1^st^ 2013 to August 31^st^ 2016, 24408 pregnant women received the first trimester Combined test and contingently offered second trimester maternal serum screening to identify those women who would most benefit from invasive prenatal diagnosis (IPD). The screening was based on first trimester cut-offs of ≥1:30 (IPD indicated), 1:31 to 1:899 (second trimester screening indicated) and ≤1:900 (no further action), and a second trimester cut-off of ≥1:250. From January 2014, analysis of fetal cells from peripheral maternal blood was also offered to women with positive screening results. For fetal Down syndrome, the overall detection rate was 96.8% for a false-positive rate of 2.8% resulting in an odds of being affected given a positive result (OAPR) of 1:11, equivalent to a positive predictive value (PPV) of 8.1%. Additional chromosome abnormalities were also identified resulting in an OAPR for any chromosome abnormality of 1:6.6 (PPV 11.9%). For a sub-set of cases with positive contingent test results, FISH analysis of circulating fetal cells in maternal circulation identified 7 abnormal and 39 as normal cases with 100% specificity and 100% sensitivity. We conclude that contingent screening using conventional Combined and second trimester screening tests is effective but can potentially be considerably enhanced through the addition of fetal cell analysis.

## Introduction

In recent years, many non-invasive prenatal testing approaches have been developed including measurement of maternal serum analytes, evaluation of specific ultrasound markers, and cell-free DNA (cf-DNA) analysis. The purpose is to better select those pregnant women who would most benefit from invasive prenatal diagnosis (IPD) [1–10]. These new approaches have allowed high detection rates and reduced invasive testing (chorionic villus sampling (CVS) and amniocentesis) and, as a consequence, reduce costs and fetal losses [1]. National policies differ considerably with respect to the strategies implemented to achieve high detection rates but also to reduce IPD utilization [7,10–15]. The Abruzzo Region, in central Italy, has regulated prenatal screening and diagnosis since 2001 with a regional law [16]. According to this act, IPD is offered to all pregnant women ≥ 35years, or with positive serologic test, irrespective of maternal age. Starting from 2012 the identification of high-risk pregnant women was based on the result of contingent screening [17–19].

Application of cf-DNA testing offers the opportunity to radically change prenatal screening but the introduction into actual clinical practice is challenging because of cost, differences in the scope of abnormalities detectable, and integration into existing testing [20–21]. Recently, the analysis of fetal cells from peripheral maternal blood has also been shown to be effective in helping to identify fetal aneuploidy. In a proof-of principle study using this approach we were able to demonstrate detection of 6/7 (86%) of aneuploid fetuses with 0/165 (0%) false-positive results [22–23]. Combination of conventional screening and fetal cell analysis therefore also offers opportunities to maximize screening efficacy while managing cost.

The goal of this study was to prospectively evaluate the efficacy of contingent screening in over 24,000 women with singleton pregnancies and to demonstrate how this can be further enhanced through the inclusion of fetal cells analysis.

## Materials and methods

This study included referrals for contingent screening between January 1^st^ 2013 to August 31^st^ 2016. The first component of the contingent prenatal screening protocol was performed between 10–13 weeks of pregnancy. Women received an ultrasound examination which included an evaluation of gestational age and nuchal translucency (NT) measurement together with measurement of maternal serum free-beta-human chorionic gonadotropin (hCG) and pregnancy associated plasma protein-A (PAPP-A). According to this testing, pregnant women were stratified in 3 categories: women with a risk of 1:900 or lower (low-risk group), women with a risk of 1:30 or higher (high-risk group), women with intermediate risk between 1:31–1:899 [4,17–19]. The result of this screening was given within 24 hours. IPD was offered to the high-risk group, low-risk pregnant women were reassured, and those with intermediate result were offered follow-up additional maternal serum screening tests between 15–17 weeks of pregnancy [24]. This consisted of alpha-fetoprotein, total hCG and unconjugated estriol measurement. All the biochemical analyses were performed with commercially available chemiluminescent immunometric assays (Siemens, Italy) at the Dept. of Medical, Oral and Biotech Sciences, Chieti University. Down syndrome risks were calculated using a dedicated software (Prisca, Siemens, Italy). Trisomy 18 [Edwards syndrome (ES)] risks were computed with the use of statistical parameters from several sources [4]. The final risk assessment of this group was based on both the first and the second trimester tests considered together and used a cut-off of 1:250 [24]. An online statistical analysis software was used for the summary measures of test performance (http://vassarstats.net/clin1.html).

Second trimester screening (triple test) was offered to women that presented too late for the first trimester screening. They were excluded from the present study.

From January 2014 we started to offer the study of fetal cells from peripheral maternal blood for the investigation of trisomies 21 and 18 to all high-risk first trimester group (>1:30) in addition to CVS. [22–23] All pregnant women who were offered this test were informed that other chromosome changes (triploidy, partial imbalances) could be incidentally detected. Moreover, this new test was associated with false positive (FP) and false negative (FN) results and all positive results needed to be confirmed by amniocentesis. After this genetic counseling and informed consent, blood samples were collected from 46 women at high risk (HR; ≥1:30) for fetal aneuploidy [24]. The average maternal age was 36.5 years (range 19–44 years), and average gestational age at sampling was 12.8 weeks (range 11–14). The average maternal body weight was 62 kg (range 46–83 kg)

Briefly, cells were isolated from maternal blood by incubation in non-physiological conditions and gradient-density separation. These cells were evaluated by FISH analysis using two differently labeled probes, specific for different loci of chromosomes 21 and 18 [22–23]. At least 2000 mononuclear cells per sample were scored (range 2000–6000) by direct visualization using an appropriate triple pass-band filter (Zeiss, Jena, Germany). The results of fetal cell analyses were given within 2 weeks in more than 90% of cases.

Fetal and neonatal outcomes were obtained from review of clinical records. Neonates underwent clinical evaluation, and neonatal karyotyping if a chromosome abnormality was suspected. Follow-up also included cases that resulted in a fetal demise or abortion that underwent fetal karyotype and post-mortem evaluation. We assumed that no further cases of trisomy 21 went undetected in our population because our Genetic Unit is the referring center for all regional prenatal and postnatal diagnoses. This retrospective review of screening and diagnostic results was deemed exempt by our instructional ethical review board (Comitato Etico delle Province di Chieti e Pescara).

## Results

**Fig 1:**
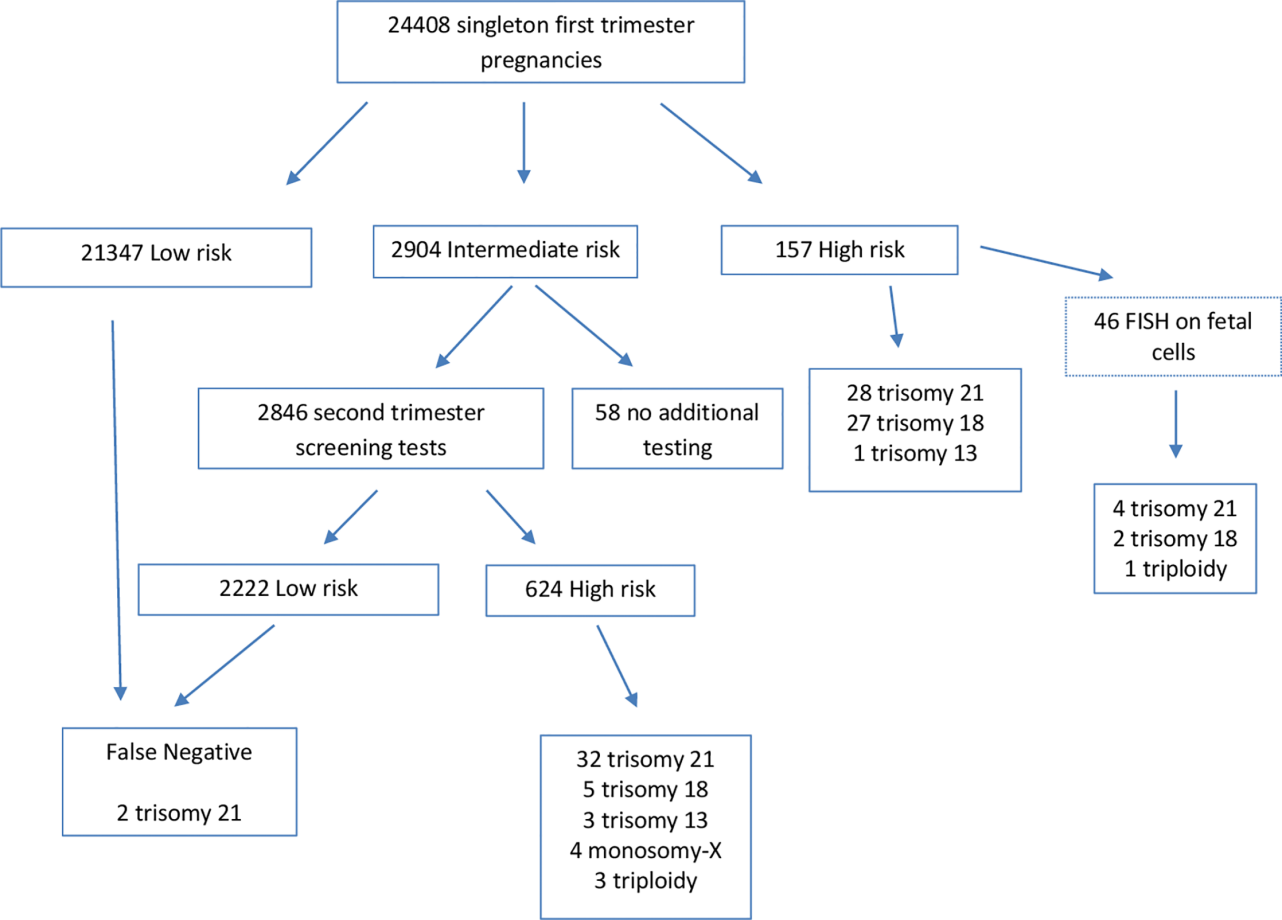
Flow diagram of cases included in the sequential screening.

This study included 24408 singleton pregnancies enrolled for contingent-based prenatal screening (Fig 1). The mean age of the women in the first trimester was 32.8 years (range 15–47 years) with 5858 (24%) age 35 years or more and 18550 (76%) under 35. There were 157 (0.64%) positive results for the testing performed in the first trimester (10–13 weeks) of which 121 were positive for DS (0.5%), and 36 for trisomy 18 (0.14%). There were 2904 (11.9%) with DS intermediate risks. Of these, 2846 received the second trimester component of the testing and at this stage 616 were DS positive and 8 trisomy 18 positive. Therefore, the total number of positive tests for DS was 737 (3%) and the total trisomy 18 positive tests was 44 (0.2%). IPD was offered and accepted in all 781 high-risk pregnancies and this showed 104 chromosome abnormalities. These chromosome changes consisted in 60 trisomy 21, 32 trisomy 18, 4 trisomy 13, 4 monosomy-X and 4 triploids all of which were ascertained through IPD with none through birth of an affected baby. There were two DS false-negative results, ascertained through livebirth affected pregnancies (1:12151). Without adjustments for fetal viability, the detection rate (DR) was therefore 96.8% (60/62 DS), and 100% (32/32) for trisomy 18 (Table 1). The false positive rate (FPR) for DS in the first trimester was 0.37%, while the total FPR for DS including the cases from the second trimester, was 2.8%. The overall rate of positive calls where no chromosome abnormality of either type was present was 2.8%. The odds of being affected having a positive result (OAPR) for trisomy 21 was 1:11 while the total OAPR for chromosome abnormality of either type was 1:6.6. Screen-positive test results were found in 476 out 5858 women aged 35 years or more who underwent IPD. After taking into consideration trisomy 13, monosomy-X, and triploidy identified in screen-positive cases, the overall OAPR for any chromosome abnormality was 1:6.5. There were 58 women received an intermediate risk based on first trimester screening but did not receive second trimester testing. There were no affected livebirths for this group.

**Table 1:** Overall, first and second trimester results of contingent screening for Down syndrome alone and for Down syndrome and trisomy 18 combined.

	First trimester	Second trimester	Net
**N**	24408	2846	24408
**Down syndrome**			
Test-positive	121/24408 (0.5%)	616/2846 (21.6%)	737/24408 (3.0%)
FPR	89/24346 (0.37%)	588/2818 (20.9%)	677/24346 (2.8%)
DR	32/62 (51.6%)	28/28 (100%)	60/62 (96.8%)
FN	2/62 (3.2%)	0/62 (0%)	2/62 (3.2%)
OAPR	1:2.8	1:21	1:11
**Down syndrome and trisomy 18 combined**			
Test-positive	157/24408 (0.6%)	624/2846 (21.9%)	781/24408 (3.2%)
FPR	98/24314 (0.4%)	591/2813 (21.0%)	689/24314 (2.8%)
DR	59/94 (62.8%)	33/33 (100%)	92/94 (97.9%)
FN	2/94 (2.1%)	0/94 (0%)	2/94 (2.1%)
OAPR	1:1.7	1:18	1:7

N = number of pregnancies; FPR = False positive rate; DR = Detection rate; FN = False negative; OAPR = Odds of being affected given a positive result. For the second trimester testing, rates are based only on the cases receiving the second trimester testing, not the total cohort.

The above data includes the patients who had the option to receive FISH analysis on fetal cells in maternal blood.

**Fig 2:**
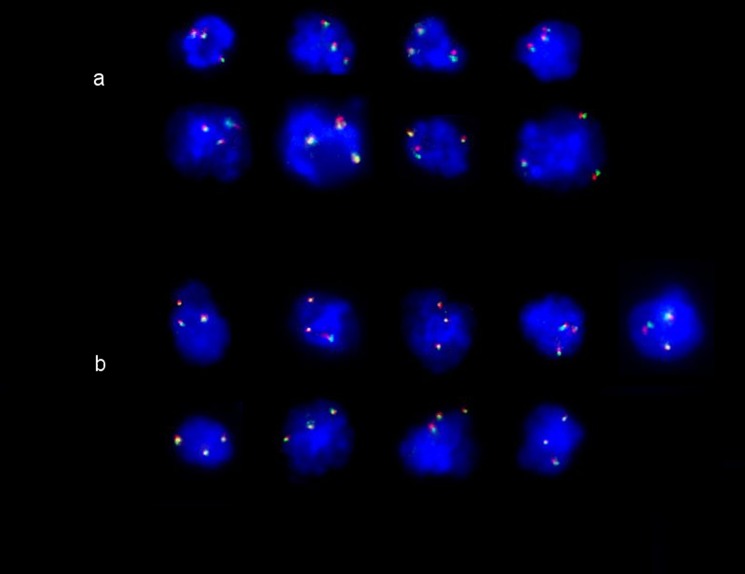
FISH signals on fetal cells in maternal blood. a) Dual-probe FISH analysis with two probes for different loci on chromosome 18 shows three signals in green and three in red in a pregnant woman with a trisomy 18 fetus; b) FISH analysis with two probes for chromosome 21 showing three signals in green and three in red specific for two different loci of chromosome 21 in a woman with a trisomy 21 fetus.

9549 women received their screening after the introduction of the FISH component of the sequential screening test and of these, 47 had a positive test result based on the conventional screening tests as described above. One woman preferred CVS while 46 accepted the analysis of fetal cells prior IPD. Results were obtained in all of the 46 fetal cell analyses. The FISH analysis disclosed 39 cases with only diploid signals for chromosomes 21 and 18 and in 7 cases extra FISH signals were present (Fig 2A and 2B). The 7 chromosome abnormalities comprised of 4 cases with trisomy 21, 2 trisomy 18 and 1 triploid. In all 46 cases, IPD confirmed either a normal karyotype or the chromosome abnormalities indicated by the FISH screening.

## Discussion

In this study, we have used a contingent screening strategy to select pregnant women who would most benefit from IPD [4,17–19]. Contingent screening utilizes two first trimester cut-offs instead of one. We have previously shown that optimal results can be obtained using a cut-off of 1:900 for low-risk and 1:30 for high-risk [19]. Using these two cut-offs, pregnant women were divided in 3 categories: women at low-risk who can be reassured and require no additional aneuploidy screening tests, those at high-risk for whom invasive testing would be recommended and those at intermediate risk who can benefit from additional second trimester screening tests. We show that this approach was effective in DS screening with an overall DR of 96.8% for a FPR of 2.8% resulting in an OAPR of 1:11 (equivalent to a positive predictive value of 8.1%). A number of other chromosome abnormalities were also identified resulting in an OAPR for any chromosome abnormality of 1:6 (PPV of 14.1%).

While this contingent test performance is impressive relative to other conventional screening test strategies, we recognize that there are also limitations. Nearly a half of the affected pregnancies are not diagnosed until the second trimester and 12% of women required second trimester screening. Non-invasive testing using cell-free DNA in maternal plasma or the identification of fetal cells in maternal circulation offer other opportunities to further improve both the efficacy and the scope of abnormalities detectable in prenatal screening [20,25]. Although substantial progress has been made in the development of tests based on the presence of fetal cf-DNA in maternal plasma, costs are high, testing generally needs to be sent to specialized referral laboratories and results are not always obtained [20,26]. The analysis of fetal cells in maternal blood has the potential to be a diagnostic test, using direct analysis of single/pooled cells. Fetal cells are amenable to FISH and/or QF-PCR analyses for rapid analysis of aneuploidies, single gene analysis, CGH-SNP analysis, or sequencing [27–29].

The presence of fetal cells in peripheral maternal blood has been known since 1969 [30]. Nevertheless, relatively little is known about the precise origin of the fetal cells or their release into maternal circulation [21–22]. Many past efforts to select for fetal cells and achieve non-invasive diagnosis for the more common chromosome aberrations showed only limited success [31–32]. We have used a fetal cell enrichment process and incorporated a FISH analysis of these cells into an existing continent screening protocol. Our preliminary results show this to be highly effective in improving the screening. Specifically, when provided this testing to 46 pregnant women where the existing protocol gave a positive result, the FISH analysis identified 7 as abnormal and 39 as normal. IPD confirmed that the fetal cell results had 100% specificity and 100% sensitivity.

Clearly, these fetal cell results need to be confirmed in a larger cohort. Because of uncertainties about the precise origin of the cells analyzed, more studies are needed to evaluate whether confined placental mosaicism can cause false-positive results. Because selection of cases was partly based on fetal findings (nuchal translucency, and fetal derived proteins) and is based on whole cells, it is possible that false-positives due to confined placental mosaicism or small copy number variants will be less common than would be the case if the test were applied to an unselected population. Cell based testing may overcome the limitations of cf-DNA in situations where fetal fraction is low. On the other hand, this cell based assay could be susceptible to erroneous results due to carry-over of cells from a previous aneuploid pregnancy or failure to detect an abnormality due to low levels of fetal cells in the maternal plasma. These aspects of the technology need further investigation.

In summary, we show that contingent based screening is an effective approach to screening. Based on our preliminary data incorporation of fetal cells in maternal serum into our contingent screening protocol has the potential to further enhance our screening performance.
